# Racemization Pathway for MoO_2_(acac)_2_ Favored over Ray–Dutt, Bailar, and Conte–Hippler Twists

**DOI:** 10.1021/acs.inorgchem.2c00824

**Published:** 2022-08-18

**Authors:** George Dhimba, Alfred Muller, Koop Lammertsma

**Affiliations:** † Department of Chemical Sciences, University of Johannesburg, Auckland Park, Johannesburg 2006, South Africa; ‡ Department of Chemistry and Pharmaceutical Sciences, Faculty of Sciences, Vrije Universiteit Amsterdam, De Boelelaan 1108, Amsterdam 1081 HZ, The Netherlands

## Abstract

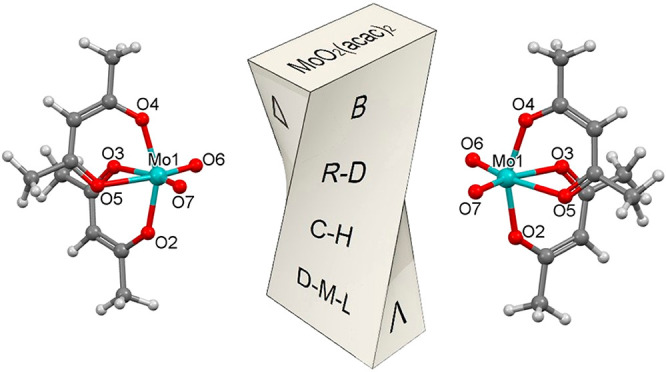

Chiral *cis*-MoO_2_(acac)_2_ racemizes via four pathways that agree with and extend upon Muetterties’ topological analysis for dynamic MX_2_(chel)_2_ complexes. Textbook Ray–Dutt and Bailar twists are the least favored with barriers of 27.5 and 28.7 kcal/mol, respectively. Rotating both acac ligands of the Bailar structure by 90° gives the lower Conte–Hippler twist (20.0 kcal/mol), which represents a valley–ridge inflection that invokes the trans isomer. The most favorable is a new twist that was found by 90° rotation of only one acac ligand of the Bailar structure. The gas-phase barrier of 17.4 kcal/mol for this Dhimba–Muller–Lammertsma twist further decreases upon inclusion of the effects of solvents to 16.3 kcal/mol (benzene), 16.2 kcal/mol (toluene), and 15.4 kcal/mol (chloroform), which are in excellent agreement with the reported experimental values.

## Abstract 2

A new pathway is presented for racemizing the Δ and Λ enantiomers of MoCl_2_(acac)_2_ that matches the reported experimental energy barrier. This Dhimba−Muller−Lammertsma (D−M−L) twist is much favored over textbook Ray−Dutt and Bailar twists and is also better than the Conte−Hippler twist. The D−M−L pathway embodies the missing link in Muetterties topological analysis of dynamic MX_2_(chel)_2_ transition-metal complexes.

Rationally designed catalysts capable of effecting enantioselective chemical transformations are crucial to satisfying the growing industrial demand for chiral fine chemicals.^1^ Despite the tremendous advances in asymmetric organocatalysis, as highlighted by the 2021 Nobel Prize in Chemistry,^2^ most catalysts used for the conversions of organic compounds are still transition-metal complexes with ligands coming from the ever-growing chiral pool.^3^ These chiral ligands are considered to be responsible for the transfer of chirality to the reaction product, but the elaborate syntheses and unpredictable enantioselectivity are limiting factors. An alternative is to solely use the stereogenicity of the metal center by the enantiomeric chelation of achiral ligands around the coordinating transition metal.^4^

Octahedral chiral complexes are, in fact, known as far back as 1911 when Werner reported on [Co(en)_3_]^3+^ (en = ethylenediamine);^5^ [Cr(en)_3_]^3+^, [Rh(en)_3_]^3+^, [Ir(en)_3_]^3+^, and [Pt(en)_4_]^4+^ were described shortly thereafter.^6^ Werner’s *D*_3*d*_-symmetrical cobalt(III) complexes carrying three simple achiral bidentate ligands were revived recently by Gladysz et al., demonstrating their effectiveness as enantioselective catalysts.^7^ In 2003, Fontecave et al. introduced the term “chiral-at-metal” catalysis and showed modest enantioselectivity for the asymmetric transfer hydrogenation and asymmetric oxidation of sulfides using [Ru(dmp)_2_(CH_3_CN)_2_]^2+^ (dmp = 2,9-dimethyl-1,10-phenanthroline).^8^ The field of chiral-at-metal catalysis was expanded majorly in the past decade by Meggers et al., who reported many different asymmetric catalytic reactions with high enantioselectivity using chiral rhodium(III) and iridium(III), [M(tbpb)_2_(CH_3_CN)_2_]^+^ (M = Rh, Ir; tbpb = 5-*tert*-butyl-2-phenylbenzoxazole),^9^ and recently with similar chiral iron(II) complexes.^10^

The asymmetric Lewis acid transition-metal complexes, carrying two bidentate and two acetonitrile ligands, apparently have high energy barriers of racemization, which enable the catalysts to maintain their chiral integrity. However, retention of chirality for other transition-metal complexes is a priori not evident because of the configurational flexibility at the metal center.^11^ Whereas such dynamics can be restricted by bi-, tri-, and tetradentate ligands, racemization is of general concern in chiral-at-metal systems. The crux is to recognize and control the dynamic pathways.

Already half a century ago, in-depth topological studies by Muetterties revealed the complexity by which penta- and hexacoordinate systems racemize.^12^ He also showed that the number of racemization pathways reduces with bidentate ligands. Illustrative is the reduction of 20 feasible permutations of a pentacoordinate system, which can be described in a Levi–Desargues graph, by introducing two bulky bidentate ligands.^13^ These cause the energy barriers for Berry pseudorotation to increase and prohibit racemization, as is the case for silicate [Si(pn)_2_F]^−^ [pn = 2-(phenyl)naphthyl].^14^ Octahedral complexes are subject to a far larger number of permutations, which also reduce upon chelation. Well-established racemization pathways for trischelate complexes are the Ray–Dutt^15^ and Bailar^16^ twists in which the chelating ligands undergo a *C*_3_ rotation^17^ via rhombic (*D*_3*h*_ symmetry) and trigonal-prismatic (*C*_2*v*_ symmetry) transition states, respectively (Figure 1).^18^ Rarer pathways include the dancing-Bailar twist,^19^ those with a bicapped tetrahedral structure,^20^ and those invoking pentacoordination.^21^ Besides Muetterties’ topological studies, little is known about the racemization pathways of octahedral complexes with two bidentate ligands, which is the subject of the present study that focuses on *cis*-MoO_2_(acac)_2_ (acac = acetylacetonate).

**Figure 1:**
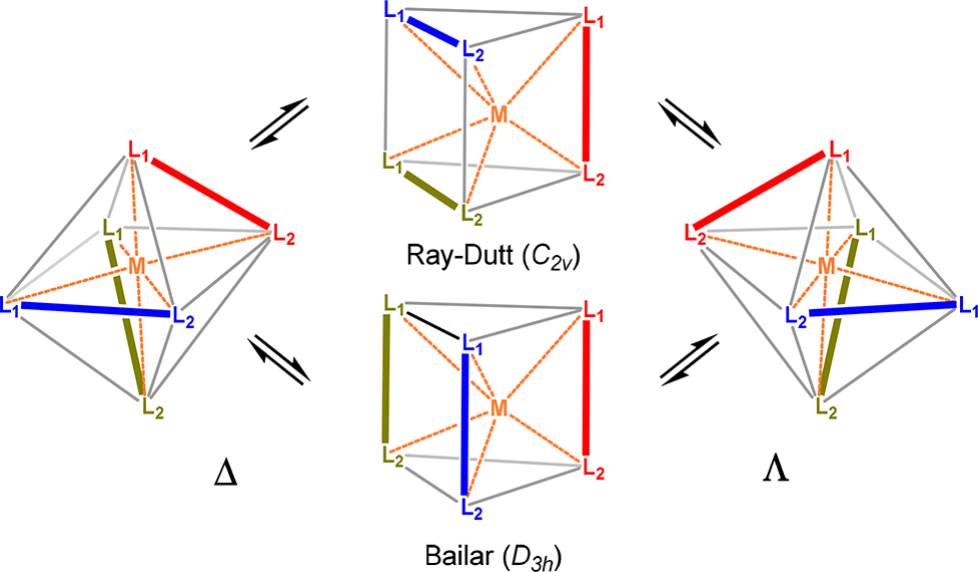
Ray–Dutt and Bailar twists by which chiral octahedral complexes undergo racemization. The three bidentate ligands are shown in blue, green, and red. The gray lines complement the edges of the octahedral and trigonal-prismatic structures, with the orange dashed lines representing the transition-metal coordination sites.

*cis*-MoO_2_(acac)_2_ is an effective catalyst for epoxidizing olefins with peroxides, but we are not aware of asymmetric homogeneous catalysis with one of its enantiomers.^22^ The solid-state structure has been reported for the racemic mixture^23^ and for an enantiomer of a derivative.^22b^ Conte and Hippler determined by variable ^1^H NMR spectroscopy a modest activation energy *E*_a_ of 16.9 kcal/mol for the racemization of *cis*-MoO_2_(acac)_2_ in benzene, 13.7 kcal/mol in chloroform, and 15.1 kcal/mol in toluene, indicating a small solvation effect.^24^ These barriers are similar to those reported by the group of Wise in 1971.^25^ SOGGA11-X/LANL2DZ+G** calculations by Conte and Hippler gave *E*_0_ barriers of 26.7 and 27.2 kcal/mol for the Ray–Dutt and Bailar twists and a lower, but still sizable, barrier of 19.4 kcal/mol for a different pathway; the heights of the barriers were not affected by inclusion of the effect of solvents. The magnitudes of these barriers seem to indicate that the racemization of *cis*-MoO_2_(acac)_2_ cannot be attributed to the Bailar or Ray–Dutt twists and likely not to the twist suggested by Conte and Hippler. Therefore, in the context of the topological analysis of MX_2_(chel)_2_ systems, we felt that a theoretical study on the racemization pathways is in order.

The potential energy surface for the MoO_2_(acac)_2_ complex was examined with *Gaussian 16*, version B01,^26^ using the hybrid meta-generalized-gradient-approximation functional ωB97X-D,^27^ which incorporates empirical dispersion terms and long-range interactions,^28^ and the 6-31G(d) basis set for C, H, and O and *LANL2DZ* for Mo.^29^ The reported absolute electronic energies for all optimized structures were estimated by single-point calculation with the 6-311+G(2d,p) basis set. Frequency and intrinsic-reaction-coordinate (IRC) calculations confirmed the nature of each transition structure.^30^ The effect of solvation was estimated by single-point calculations with the polarizable continuum solvent model at 25 °C.^31^

The geometries of Λ- and Δ-*cis*-MoO_2_(acac)_2_, shown in Figure 2, have a distorted octahedral arrangement in which the planes formed by the acac ligands and metal center are each tilted by 10.8° from orthogonality with the MoO_2_ plane. The Mo=O bonds of the MoO_2_ fragment have a length of 1.692 Å and an angle of 104.6°. The two Mo–O bonds of each acac ligand are longer and unequal to each other, i.e., 2.019 Å (Mo–O_cis_) and 2.252 Å (Mo–O_trans_), because of the different chemical environments of the two acac oxygen atoms. The methyl groups of the acac ligands are eclipsed with the methine hydrogen atoms. The geometry of Λ/Δ-*cis*-MoO_2_(acac)_2_ compares well with those of the reported X-ray crystal structures.^23^

**Figure 2:**
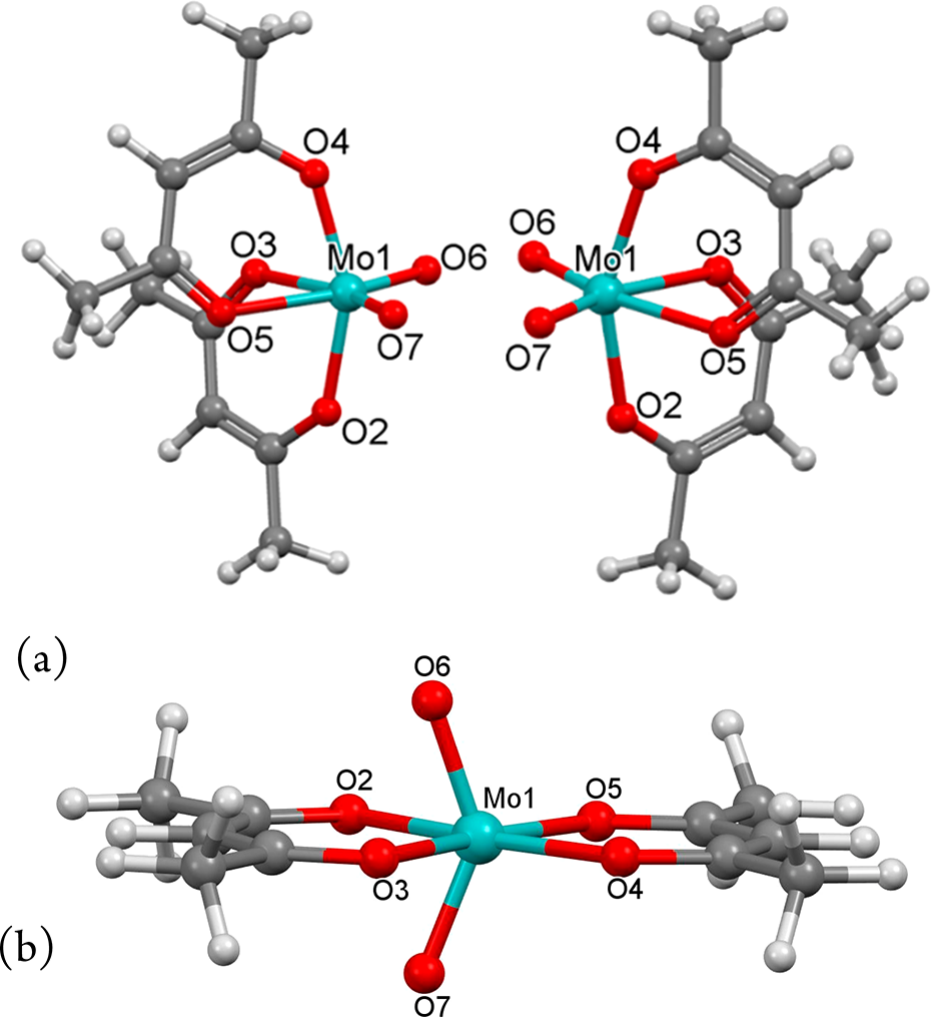
(a) Δ and Λ enantiomers of *cis*-MoO_2_(acac)_2_ and (b) *trans*-MoO_2_(acac)_2_.

*trans*-MoO_2_(acac)_2_ (*C*_2*v*_ symmetry), shown in Figure 2b, is a substantial 50.6 kcal/mol less stable than the cis isomer. It is then not surprising that no solid-state structure is known for this isomer. Moreover, geometry optimization with the extended basis set suggests it to be a transition structure (*C*_2*v*_ symmetry) at a flat energy plateau with an imaginary frequency of a mere −12 cm^–1^. The trans Mo=O bonds of its MoO_2_ fragment are longer (1.731 Å) than those in the cis isomer and deviate substantially from linearity (140.0°), and both tilt toward one of the acac ligands, which has as a result longer Mo–O bonds (2.137 Å) than the other ligand (2.034 Å).

To understand the racemization of *cis*-MoO_2_(acac)_2_ and the potential role of the trans isomer, it is instructive to analyze their topological relationship. Muetterties showed that a metal complex with six different (monodentate) ligands has 30 octahedral isomers and 120 trigonal-prismatic iosomers but that this reduces significantly for complexes with two symmetrical bidentate ligands, MX_2_(chel)_2_. Figure 3, adapted from the original study, gives the topological representation, showing the enantiomeric cis isomers at the base and the trans isomer at the apex of an isosceles triangle (open dots). The closed dots at the edges of the triangle are the trigonal-prismatic structures (Figure 3, right), embodying rearrangement of the octahedral structures.

**Figure 3:**
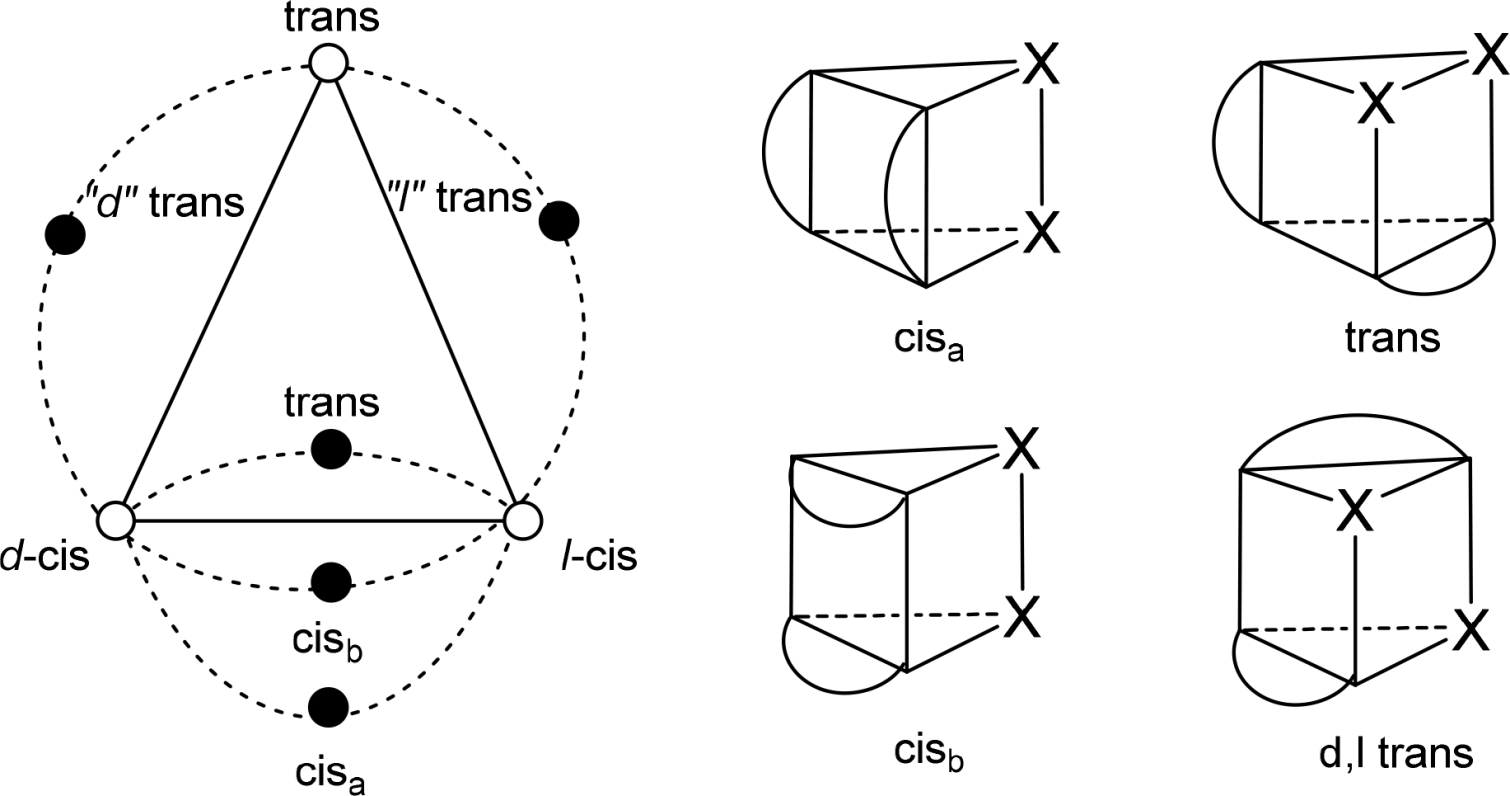
Topological representation of MX_2_(chel)_2_ with octahedral structures (open dots) and connecting trigonal-prismatic structures (closed dots) shown separately.

Topological analysis gives three direct racemization pathways for *cis*-MX_2_(chel)_2_, each with a trigonal-prismatic transition structure (cis_a_, cis_b_, and trans in Figure 3), complemented by a pathway via the trans isomer that involves a set of enantiomeric structures (d,l trans). We are unaware whether all of these racemization pathways have found solid footing in the literature. Consequently, we felt MoO_2_(acac)_2_ was ideal to verify topological analysis in the context of comparing the racemization barriers of the cis isomer with the reported experimental one.

The obvious places to start with are the established Ray–Dutt and Bailar twists for trischelating octahedral systems (Figure 1), which are represented respectively as cis_b_ and cis_a_ in Figure 3. Their corresponding *C*_2*v*_-symmetric transition structures for MoO_2_(acac)_2_ (Figure 4) have relative energies of a significant 27.5 and 28.7 kcal/mol, respectively. The structure for the Ray–Dutt twist has its MoO_2_ unit (*d*_Mo=O_ = 1.696 Å; ∠_OMoO_ = 97.4°) bisecting both virtually planar acac ligands (*d*_Mo=O_ = 2.128 Å), which have an intercept angle of 19.6°. In the Bailar transition structure, the MoO_2_ unit (*d*_Mo=O_ = 1.687 Å; ∠_OMoO_ = 95.8°) is rotated by 90° and has a larger bisecting angle of 48.3° between the acac ligands (*d*_Mo=O_ = 2.148 Å).

**Figure 4:**
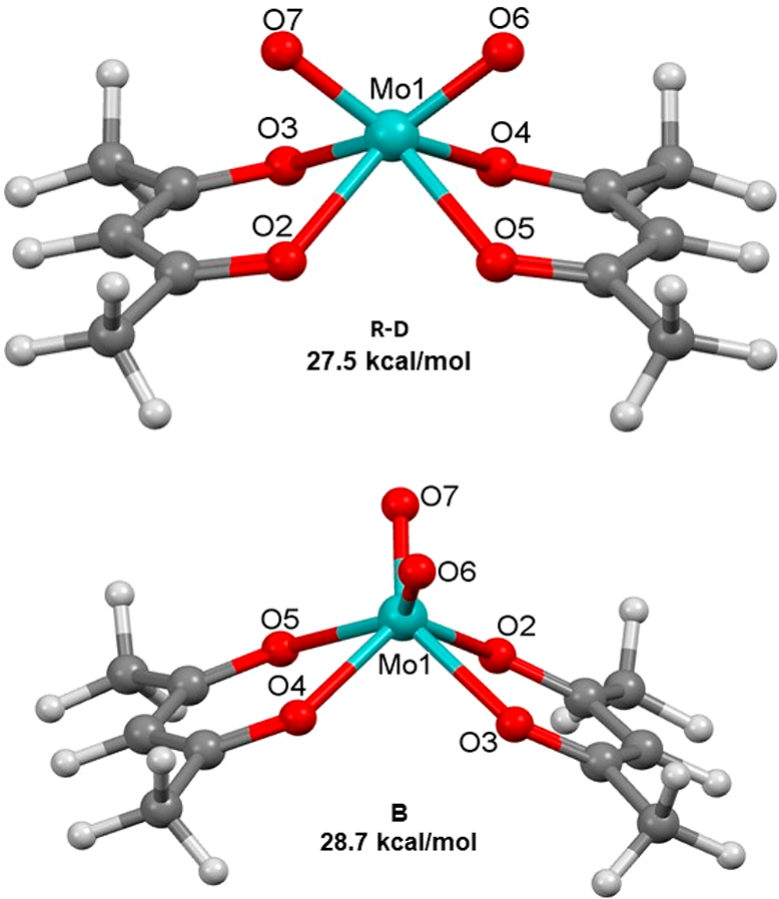
Ray–Dutt (top) and Bailar (bottom) transition structures for the racemization of *cis*-MoO_2_(acac)_2_.

Next, we focus on the role of *trans*-MoO_2_(acac)_2_ in isomerization of the cis isomer and on how the d,l trans forms (Figure 3) are involved. The latter can be considered to result from the Bailar transition structure by 90° rotation of both acac ligands. Such a transformation gives indeed a transition structure (Figure 5) with a relative energy of 20.0 kcal/mol, akin to that reported by Conte and Hippler.^31^ The two planar acac ligands of the *C*_2*v*_-symmetric structure lie in the same plane, with each having Mo–O bonds of 2.040 and 2.264 Å to the MoO_2_ unit (*d*_Mo=O_ = 1.693 Å; ∠_OMoO_ = 117.2°). The IRC confirms that this transition structure is yet another structure for the racemization of *cis*-MoO_2_(acac)_2_ (see the Supporting Information) by opposite rotation of the acac ligands, but it still does not match the reported experimental value.

**Figure 5:**
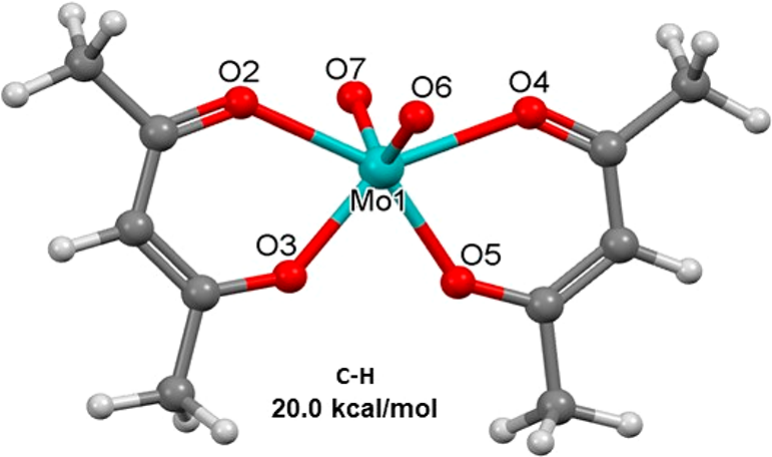
Conte–Hippler transition structure for the racemization of *cis*-MoO_2_(acac)_2_.

Further inspection of the *C*_2*v*_-symmetric structure is revealing. Rotating the MoO_2_ plane that bisects the two acac ligands by 90° and enlarging the O=Mo=O angle (117.2° → 140.0°) results in *C*_2*v*_-symmetric *trans*-MoO_2_(acac)_2_ (Figure 2b). This rotation can be left- or right-handed so that the MoO_2_ unit gets directed toward either one or the other acac ligand, which is in accordance with topological analysis (Figure 3). The high-energy trans isomer lies on a very flat high-energy plateau that allows for slight bending of its acac ligands. Despite the technical difficulties that this caused, we obtained an IRC that connects *trans*-MoO_2_(acac)_2_ by left- and right-handed rotation of the MoO_2_ unit to the Conte–Hippler transition structure (Figure 5) and thus ultimately to Δ- and Λ-*cis*-MoO_2_(acac)_2_. Evidently, this transition structure is a valley–ridge inflection point that gives one *cis*-MoO_2_(acac)_2_ enantiomer when the IRC is followed in one direction, likely because of torque selectivity.^32^ The relationship is shown in a simplified manner in Figure 6.

**Figure 6:**
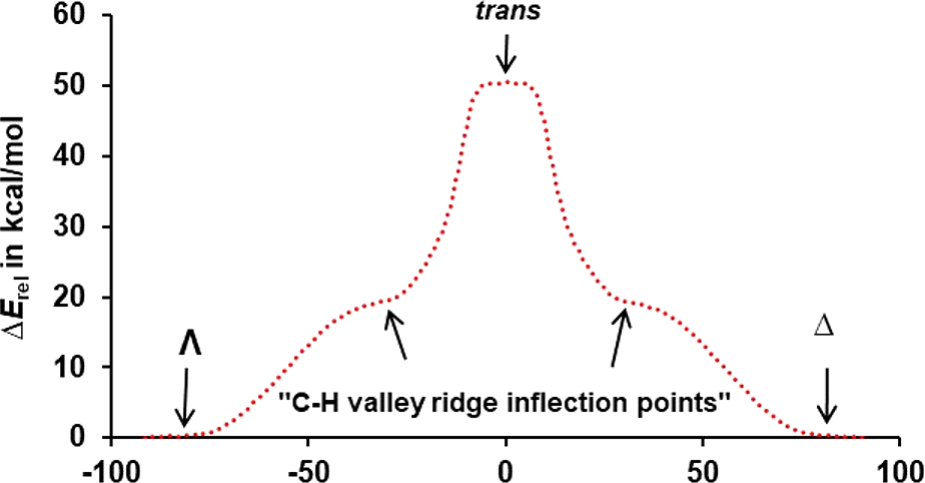
IRC for *trans*-MoO_2_(acac)_2_.

The only remaining racemization pathway to consider is that of the trans form in Figure 3. This twist is readily conceived by rotating one of the chelates of cis_a_ by 90° instead of both. Such a rotation of one acac ligand of the Bailar structure led, in fact, to the hitherto unknown transition structure shown in Figure 7. Tracing the IRC trajectory confirms that it represents a new racemization pathway for *cis*-MoO_2_(acac)_2_ (see Figure S1). The two planar acac ligands of the structure lie in the orthogonal planes, with one having two symmetrical *d*_Mo=O_ bonds (2.176 Å) and the other two unsymmetrical bonds (2.108 and 2.120 Å) to the MoO_2_ unit (*d*_Mo=O_ = 1.687 Å; ∠_OMoO_ = 101.1°). Most importantly, this new transition structure reflects the lowest-energy barrier for the racemization of *cis*-MoO_2_(acac)_2_ with a barrier of only 17.4 kcal/mol and on including the effects of solvation of 16.3 kcal/mol (benzene), 16.2 kcal/mol (toluene), and 15.4 kcal/mol (chloroform). The calculated barriers for these different solvent systems compare exceptionally well with the experimental *E*_a_ values of 16.9 kcal/mol (benzene), 15.1 kcal/mol (toluene), and 13.7 kcal/mol (chloroform), which were determined by Conte and Hippler.^24^ Evidently, this new twist represents the most favorable pathway by which the enantiomers of *cis*-MoO_2_(acac)_2_ racemize.

**Figure 7:**
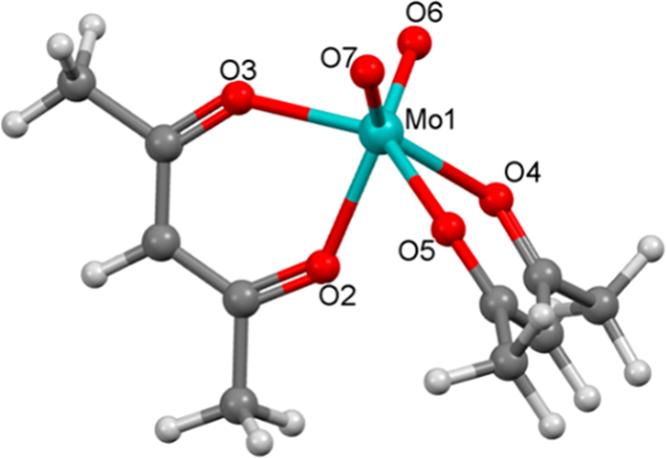
D–M–L transition structure for the racemization of *cis*-MoO_2_(acac)_2_.

In conclusion, the four pathways by which Δ- and Λ-*cis*-MoO_2_(acac)_2_ can racemize are the Ray–Dutt and Bailar twists and those in which one or both chelates of the Bailar twist are rotated by 90° (Figure 8). The barrier of 17.4 kcal/mol for the pathway with one rotated acac ligand, the Dhimba–Muller–Lammertsma (D–M–L) twist, agrees excellently with that determined experimentally. The less favored C–H twist in which both acac ligands are rotated represents a valley–ridge inflection that invokes the trans isomer. The well-established Ray–Dutt and Bailar twists are by far the least favored pathways. The obtained results agree fully with Muetterties’ topological analysis and give confidence that they apply to all dynamic MX_2_(chel)_2_ complexes. Inhibiting racemization of such complexes with properly substituted bidentate ligands can propel asymmetric catalysis with chiral-at-metal catalysts derived from readily available, inexpensive transition metals, which we are currently exploring.

**Figure 8:**
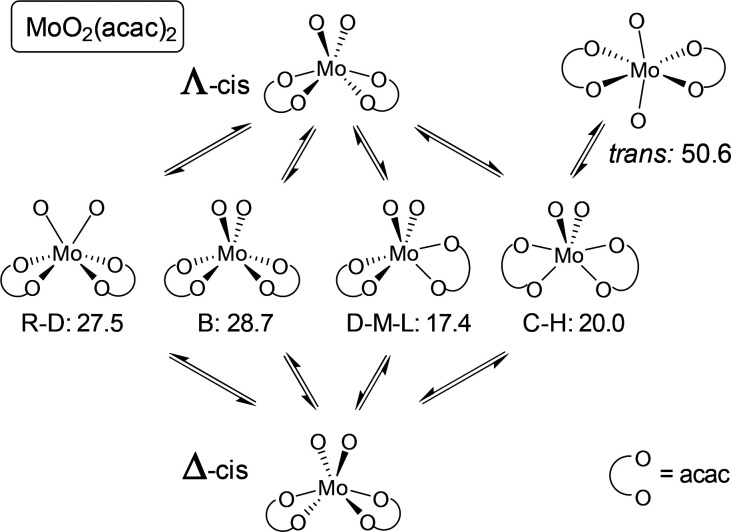
Schematic presentation of the racemization pathways for (nonsolvated) *cis*-MoO_2_(acac)_2_ with relative energies (kcal/mol) for the Ray–Dutt (R–D), Bailar (B), Dhimba–Muller–Lammertsma (D–M–L), and Conte–Hippler (C–H) transition structures and the trans isomer.

## Supporting Information Available

All computational details (PDF)
