# Supplementary material for: Racemization Pathway for MoO2(acac)2 Favored over Ray–Dutt, Bailar, and Conte–Hippler Twists
Source: Inorg Chem. 2022 Aug 18;61(38):14918–23. doi: 10.1021/acs.inorgchem.2c00824 (PMC9516665; doi:10.1021/acs.inorgchem.2c00824)
Supplement: Supplementary file 1 — ic2c00824_si_001.pdf [file ic2c00824_si_001.pdf]

Supporting Information

A Racemization Pathway for $MoO_2(acac)_2$ favored over Ray-Dutt, Bailar, and Conte-Hippler Twists

George Dhimba,[a] Alfred Muller,[a] and Koop Lammertsma[a,b,*]

[a] Department of Chemical Sciences, University of Johannesburg, Auckland Park, Johannesburg, 2006, South Africa

[b] Department of Chemistry and Pharmaceutical Sciences, Faculty of Sciences, Vrije Universiteit Amsterdam, De Boelelaan 1108, 1081 HZ Amsterdam, The Netherlands

corresponding e-mail: k.lammertsma@vu.nl

A. Geometries

Δ-*cis*-$MoO_2(acac)_2$ (C_2, $N_{imag} = 0$)

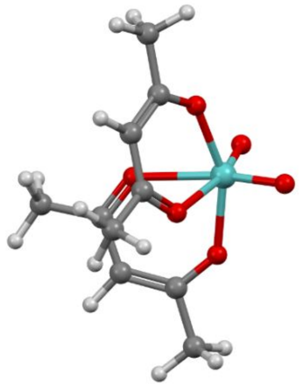

Atom	X	Y	Z
Mo	0.004898	-0.008609	-1.705799
O	1.645857	1.080711	-1.261452
O	0.746457	-1.144212	0.092801
O	-1.637455	-1.097847	-1.266543
O	-0.742429	1.127252	0.090236
O	0.698321	-1.155306	-2.739607
O	-0.685138	1.137940	-2.742019
C	3.388637	2.324168	-0.325780
C	2.424224	1.175092	-0.233625
C	2.424078	0.329038	0.857664
C	1.599318	-0.819685	0.953437
C	1.741503	-1.743783	2.137256
H	3.989915	2.217344	-1.234214
H	2.824095	3.257607	-0.416863
H	4.048790	2.379681	0.542032
H	3.134135	0.522412	1.651948
H	2.411536	-1.349026	2.904111
H	0.751633	-1.923496	2.567374
H	2.125440	-2.708442	1.788922
C	-1.598037	0.802867	0.948188
C	-2.419153	-1.192069	-0.241226
C	-1.744128	1.727232	2.131322
C	-2.422530	-0.345843	0.849923
C	-3.383188	-2.341224	-0.336280
H	-2.127096	2.691753	1.781537
H	-2.416553	1.332562	2.896126
H	-0.755662	1.907191	2.564550
H	-3.135183	-0.539053	1.641917
H	-4.046074	-2.396670	0.529450
H	-3.981604	-2.234544	-1.246619
H	-2.818305	-3.274639	-0.425468

trans-$MoO_2(acac)_2$ (C_{2v}, $N_{imag,}$= 1: 12.43*i* cm^{-1})

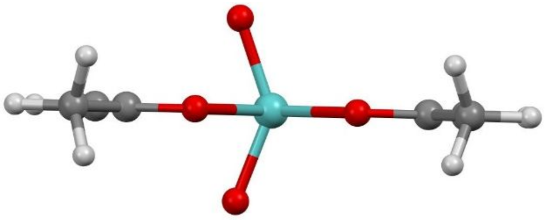

Atom	X	Y	Z
Mo	0.000000	0.000000	0.004588
O	0.000000	1.639565	-1.352289
O	0.000000	-1.639565	-1.352289
O	0.000000	-1.331759	1.553407
O	0.000000	1.331759	1.553407
O	-1.625856	0.000000	-0.587384
O	1.625856	0.000000	-0.587384
C	0.000000	0.000000	-3.143275
H	0.000000	0.000000	-4.226400
C	0.000000	0.000000	3.499790
H	0.000000	0.000000	4.581803
C	0.000000	-1.297319	-2.581428
C	0.000000	1.297319	-2.581428
C	0.000000	1.223370	2.827888
C	0.000000	-1.223370	2.827888
C	0.000000	2.477526	-3.525309
H	0.000000	2.180851	-4.575651
H	-0.882412	3.092532	-3.323019
H	0.882412	3.092532	-3.323019
C	0.000000	-2.477526	-3.525309
H	-0.882412	-3.092532	-3.323019
H	0.000000	-2.180851	-4.575651
H	0.882412	-3.092532	-3.323019
C	0.000000	-2.525668	3.572103
H	0.881350	-3.103921	3.277229
H	0.000000	-2.383569	4.654223
H	-0.881350	-3.103921	3.277229
C	0.000000	2.525668	3.572103
H	0.000000	2.383569	4.654223
H	0.881350	3.103921	3.277229
H	-0.881350	3.103921	3.277229

R-D (C_{2v}, N_{imag} = 1: 109.11i cm^{-1})

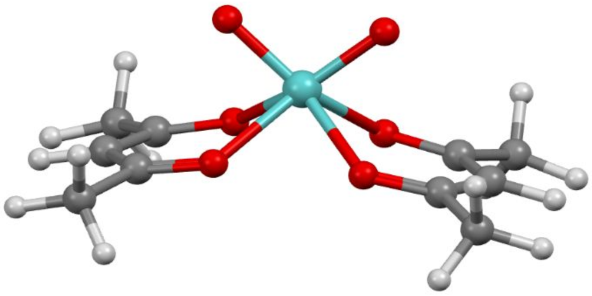

Atom	X	Y	Z
Mo	0.020004	0.368398	0.036899
O	1.754130	-0.686181	-0.602810
O	0.384560	-0.713873	1.832602
O	-1.737484	-0.669104	0.639710
O	-0.367905	-0.641427	-1.795725
O	-1.077820	1.511590	-0.567559
O	1.142826	1.464772	0.680873
C	4.042922	-1.203404	-0.598425
C	2.756105	-0.948207	0.140987
C	2.716978	-1.045069	1.537530
C	1.543142	-0.972711	2.297897
C	1.582477	-1.252765	3.777010
H	3.873845	-1.968815	-1.361687
H	4.336402	-0.284706	-1.117185
H	4.853109	-1.517464	0.062764
H	3.635749	-1.300177	2.051315
H	2.570720	-1.565711	4.120009
H	1.285656	-0.344977	4.312830
H	0.847913	-2.027399	4.016451
C	-1.531796	-0.857771	-2.269401
C	-2.744752	-0.882338	-0.112477
C	-1.576812	-1.084364	-3.757480
C	-2.707237	-0.930773	-1.511583
C	-4.037288	-1.134454	0.617960
H	-0.859352	-1.865963	-4.024546
H	-2.571584	-1.363187	-4.110884
H	-1.259975	-0.164899	-4.260955
H	-3.631269	-1.147051	-2.033680
H	-4.853621	-1.408464	-0.053402
H	-3.885313	-1.928543	1.355060
H	-4.311228	-0.227562	1.167301

B (C_{2v}, N_{imag} =1: 105.53i cm^{-1})

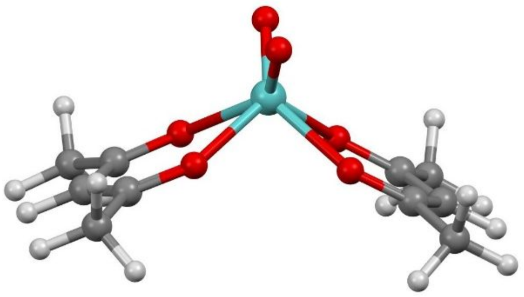

Atom	X	Y	Z
Mo	-0.486844	-0.157429	2.013991
O	1.499087	0.396606	1.413058
O	0.610129	-1.998629	1.877707
O	-1.859961	-1.288805	0.811272
O	-0.971000	1.106421	0.346593
O	-1.263370	-0.983760	3.262531
O	-0.406562	1.324846	2.814671
C	3.784913	0.811482	1.073076
C	2.655450	-0.133340	1.387208
C	2.893506	-1.489506	1.614382
C	1.826971	-2.365623	1.820245
C	2.056552	-3.845466	1.976481
H	3.590544	1.295075	0.110701
H	3.811524	1.599349	1.832414
H	4.752331	0.305680	1.037786
H	3.906182	-1.870626	1.587165
H	3.112317	-4.113208	1.894911
H	1.672492	-4.164353	2.950568
H	1.484358	-4.379682	1.211618
C	-1.859579	1.164146	-0.562135
C	-2.688060	-1.068130	-0.129069
C	-1.868525	2.436119	-1.367776
C	-2.765550	0.136756	-0.828863
C	-3.596880	-2.220822	-0.464333
H	-0.882653	2.580575	-1.820543
H	-2.630848	2.427393	-2.149930
H	-2.043769	3.281973	-0.695572
H	-3.503015	0.258588	-1.611682
H	-4.270925	-1.991430	-1.292597
H	-2.988766	-3.094185	-0.719764
H	-4.182778	-2.481780	0.422606

C-H (C_{2v}, N_{imag} = 1: 23.5*i* cm^{-1})

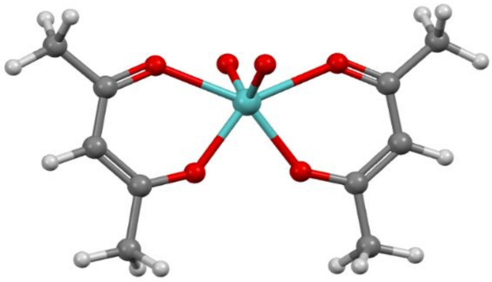

Atom	X	Y	Z
Mo	-0.000311	-0.432505	0.058345
O	-2.088744	-1.305173	0.119216
O	-1.216203	1.201680	-0.060053
O	2.087479	-1.306526	0.122549
O	1.216831	1.200839	-0.058861
O	0.000421	-1.415907	-1.320271
O	-0.001684	-1.209750	1.562750
C	-3.501777	0.574272	-0.021120
H	-4.526943	0.920207	-0.048715
C	3.501936	0.571945	-0.016450
H	4.527366	0.917202	-0.042696
C	-4.382607	-1.796726	0.154045
H	-5.356732	-1.308656	0.077170
H	-4.322395	-2.340853	1.101791
H	-4.275099	-2.530153	-0.650579
C	4.380975	-1.799575	0.160643
H	4.274823	-2.532305	-0.644807
H	4.318353	-2.344412	1.107816
H	5.355547	-1.312014	0.086237
C	2.750956	2.963266	-0.187314
H	2.284509	3.349652	-1.099169
H	3.819852	3.185969	-0.203407
H	2.284509	3.476328	0.659755
C	-2.748986	2.965159	-0.190070
H	-2.283423	3.477619	0.657847
H	-3.817712	3.188568	-0.207623
H	-2.280973	3.351554	-1.101118
C	-3.238847	-0.816236	0.080812
C	-2.480385	1.489693	-0.083872
C	2.481235	1.488014	-0.080987
C	3.237955	-0.818359	0.085591

D-M-L (C_{2v}, N_{imag} = 1: 85.14*i* cm^{-1})

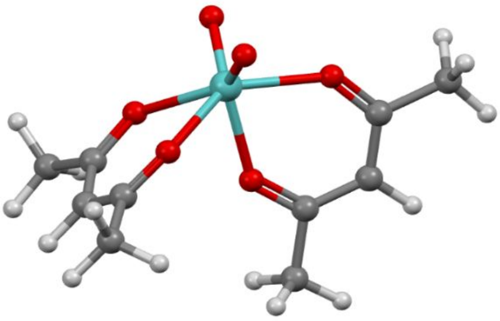

Atom	X	Y	Z
Mo	0.059729	0.158457	1.458679
O	0.830414	0.162760	-0.515824
O	2.140895	0.249774	1.780714
O	-1.243113	-1.238338	0.416058
O	-1.352688	1.414163	0.377706
O	0.011944	-1.132718	2.544012
O	-0.097192	1.471238	2.506937
C	1.968737	0.182361	-2.566364
C	1.981221	0.203028	-1.062501
C	3.175664	0.262132	-0.348900
C	3.181821	0.282009	1.047792
C	4.475998	0.345792	1.811950
H	1.459661	-0.725012	-2.906395
H	1.387523	1.035752	-2.929766
H	2.973695	0.217962	-2.991700
H	4.116344	0.293481	-0.882767
H	5.345162	0.371530	1.151610
H	4.471854	1.237038	2.447055
H	4.543424	-0.524077	2.472601
C	-2.085781	1.255769	-0.647846
C	-1.985513	-1.170202	-0.612998
C	-2.645071	2.528651	-1.231196
C	-2.402762	0.019183	-1.211966
C	-2.436644	-2.501099	-1.159301
H	-1.818488	3.197582	-1.490721
H	-3.259660	2.347721	-2.115978
H	-3.245363	3.036708	-0.469860
H	-3.053019	-0.020131	-2.076719
H	-3.064831	-2.396863	-2.046893
H	-1.557072	-3.105316	-1.402979
H	-2.991580	-3.036463	-0.382457

B. Energies.

Table S1. Energies *E*, *ZPVE* energies, enthalpies *H*, and free energies *G* (all in a.u.) and their differences Δ (in kcal/mol) for the transition structures optimized at ωB97XD/(6-31G(d) (LanL2DZ for Mo) relative to that of the Δ enantiomer of *cis*-$MoO_2(acac)_2$; *nf* = number of imaginary frequencies.

	E	Δ	E_{ZPVE}	Δ	*H*	Δ	*G*	Δ	*nf*
Λ-enantiomer	-908.2062	0.0	-907.9702	0.0	-907.9491	0.0	-908.0201	0.0	0
R-D	-908.1625	27.4	-907.9257	27.9	-907.906	27.0	-907.9733	29.4	1
B	-908.1622	27.6	-907.9262	27.6	-907.9059	27.1	-907.9751	28.2	1
C-H	-908.1745	19.9	-907.9385	19.9	-907.9181	19.5	-907.9893	19.3	1
D-M-L	-908.1795	16.8	-907.9435	16.7	-907.9231	16.3	-907.9930	17.0	1
trans isomer	-908.1251	50.9	-907.889	50.8	-907.8691	50.2	-907.939	50.9	1

Table S2. Single point energy calculation at ωB97XD/(6-311+G(2d,p) //(6-31G(d) (LanL2DZ for Mo). Energies are in a.u. and relative energies in kcal/mol.

Structure	*E*	Δ
Λ-enantiomer	-908.4733	0.0
R-D	-908.4295	27.5
B	-908.4276	28.7
C-H	-908.4414	20.0
D-M-L	-908.4456	17.4
trans	-908.3926	50.6

Table S3. PCM solvation effects on the D-M-L twist. Energies in a.u. and relative energies in kcal/mol at ωB97XD/(6-311+G(2d,p) (LanL2DZ for Mo).

Solvent		*E*	Δ
Benzene	Λ-enantiomer	-908.480404	
	D-M-L	-908.454500	16.3
$CHCl_3$	Λ-enantiomer	-908.484756	
	D-M-L	-908.460207	15.4
Toluene	Λ-enantiomer	-908.480725	
	D-M-L	-908.454915	16.2
CH_3CN	Λ-enantiomer	-908.489508	
	D-M-L	-908.466638	14.4

C. Intrinsic Reaction Coordinates.

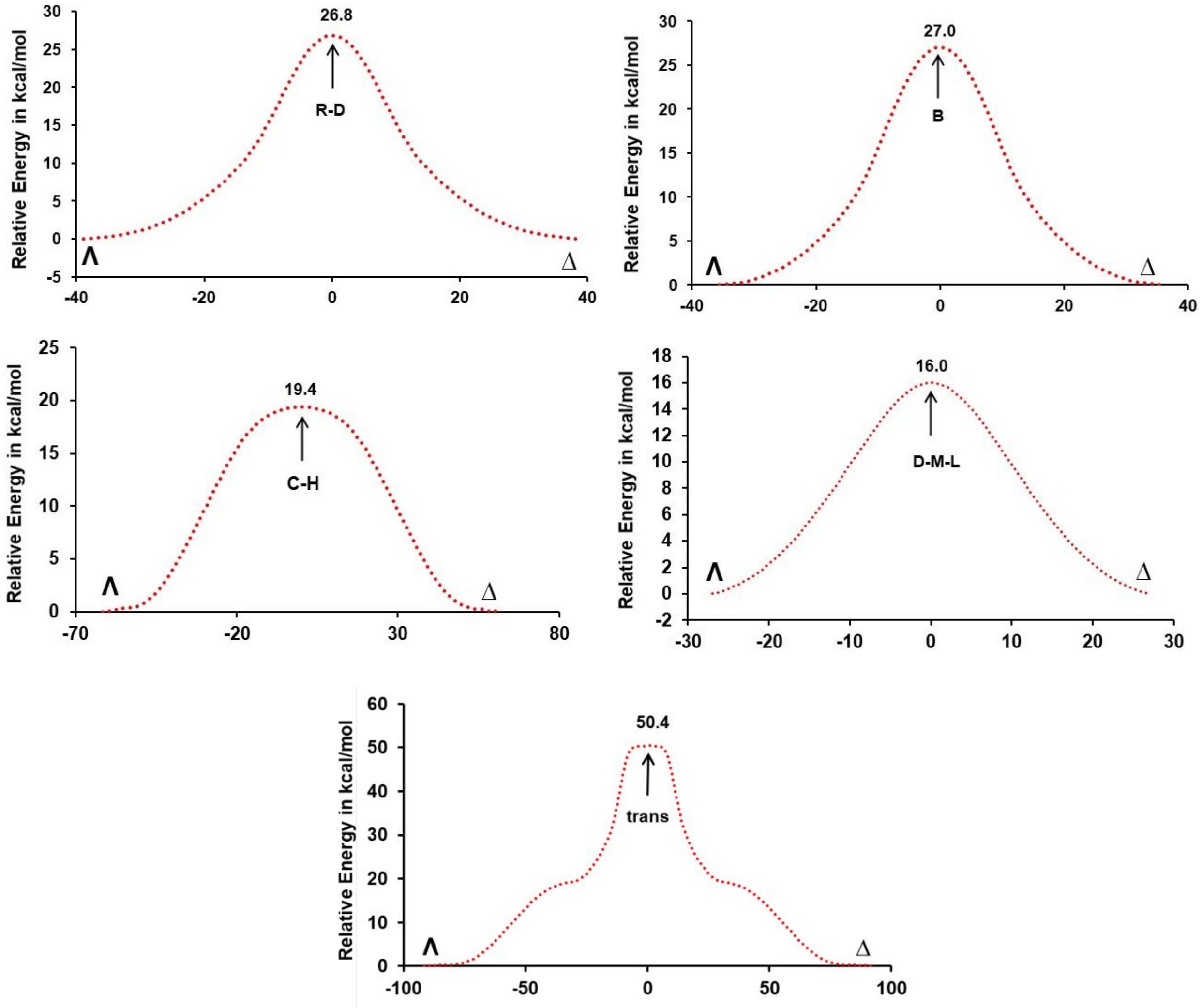

Figure S1. IRCs at ωB97XD/(6-31G(d) (LanL2DZ for Mo) for racemization of *cis*-$MoO_2(acac)_2$. through four non dissociation twisting mechanisms discussed in this work.

The IRC scans were performed in both directions for the transition structures of the Ray-Dutt (R-D), Bailar (B), Conte-Hippler (C-H), and Dhimba-Muller-Lammertsma (D-M-L) twists. Each IRC gave smoots connections to the Λ and Δ enantiomers of the *cis*-$MoO_2(acac)_2$. complex. The calculations were performed within the Gaussian 16 suite of programs using the keyword ‘calcall’ so that the force constants were calculated at each step to ensure that the IRC path was followed properly. The very flat potential energy surface around the C-H and *trans*-$MoO_2(acac)_2$ transition structures necessitated the use of the keyword ‘nogradstop’.
